# A case report of congenital erythropoietic anemia II in China with a novel mutation

**DOI:** 10.1007/s00277-019-03612-2

**Published:** 2019-02-12

**Authors:** Hong Zhang, Wuqing Wan, Xiaoyan Liu, Chuan Wen, Ying Liu, Senlin Luo, Xiao Sun, Shizhe Liu

**Affiliations:** Department of Pediatrics, the Second Xiangya Hospital, Central South University, Changsha, 410011 Hunan China

Dear Editor,

Congenital erythropoietic anemias (CDAs) are a group of rare inherited diseases [1]. So far, the CDAs are mainly divided into four types (type I to type IV), and the CDA type II is the most common type. It is caused by a mutation in the SEC23B gene. To date, 67 causative mutations in the SEC23B gene have been described [2–5] (the complete mutational spectrum of *SEC23B* is shown in Table 1).

**Table 1:** Mutational spectrum of *SEC23B*

Exon	Nucleotide change	AA change
Missense mutations		
2	c.40C > T	R14W
2	c.53G > A	R18H
2	c.74C > A	P25H
2	c.197G > A	C66Y
4	c.325G > A	E109K
5	c.494G > A	G165D
7	c.716A > G	D239G
8	c.938G > A	R313H
8	c.953T > C	I318T
9	c.1043A > C	D348A
10	c.1157A > G	Q386R
11	c.1254T > G	I418M
11	c.1307C > T	S436L
11	c.1352G > T	C451F
12	c.1385A > G	Y462C
12	c.1445A > G	Q482R
13	c.1453A > G	T485A
13	c.1467C > G	H489Q
13	c.1489C > T	R497C
13	c.1508G > A	R503Q
14	c.1571C > T	A524V
14	c.1588C > T	R530W
14	c.1589G > A	R530Q
14	c.1654C > T	L552F
15	c.1685A > G	Y562C
15	c.1727T > C	F576S
15	c.1733T > C	L578P
15	c.1735T > A	Y579N
16	c.1808C > T	S603L
16	c.1832G > C	R611P
16	c.1858A > G	M620V
16	c.1859T > C	M620T
17	c.1910T > G	V637G
17	c.1949T > C	L650S
17	c.1968T > G	F656L
18	c.2101C > T	R701C
17	c.2108C > T	P703L
18	c.2129C > T	T710M
19	c.2166A > C	K723Q
19	c.2180C > T	S727F
20	c.2270A > C	H757P
Nonsense mutations		
2	c.71G > A	W24X
3	c.235C > T	R79X
5	c.367C > T	R123X
5	c.568C > T	R190X
6	c.640C > T	Q214X
6	c.649C > T	R217X
7	c.790C > T	R264X
8	c.970C > T	R324X
9	c.1015C > T	R339X
10	c.1201C > T	R401X
14	c.1603C > T	R535X
14	c.1648C > T	R550X
14	c.1660C < T	R554X
Splicing mutations		
2–3	c.221 + 31A > G	
3–4	c.279 + 3A > G	
6	c.689 + 1G > A	
9–10	c.1109 + 1G > A	
9–10	c.1109 + 5G > A	
18–19	c.2149-2A > G	
Frameshift mutations		
3	c.222-817_366 + 4242del	
5	c.387(delG)	
5	c.428_428delAinsCG	
9	c.1063(delG)	
16	c.1821delT	
17	c.1962-64(delT)	
19	c.2150(delC)	
Small deletion		
16	c.1857_1859delCAT	

We report a patient with typical clinical manifestations and laboratory findings, a 6-year-old girl who had suffered jaundice at the age of 6 months with low hemoglobin levels at 80 g/L. Her hemoglobin concentration fluctuated between 80 and 100 g/L, and the severe hemoglobin lows were complicated with jaundice, which was not treated. There was no clear diagnosis even after comprehensive examinations. She tended to catch colds easily. Her parents and a younger brother were all healthy. Upon physical examination, the proband displayed anemic facies and yellowish discoloration of the mucous membrane and skin. Abdominal examination showed hepatomegaly and splenomegaly.

Laboratory investigations showed a hemoglobin level of 78 g/L. Reticulocyte count was 0.069 × 10^12/L, and reticulocyte ratio was 3.16%. Total bilirubin was 53.9 μmol/L (normal, 0–21), of which 42.7 μmol/L was indirect (normal, 0–19). G6PD deficiency was not found. Red blood cell folate and hemoglobin electrophoresis gave results within normal limits. Serum vitamin B12 was 736 pmol/L (normal, 133–675). Serum iron, ferritin, and transferrin were all within normal limits. Erythrocyte osmotic fragility test was normal. Acidified glycerol hemolysis test and Coombs test were negative. Light microscope observation of a bone marrow smear revealed hyperplasia and binucleated late erythroblasts (Fig. 1a).

**Fig. 1:**
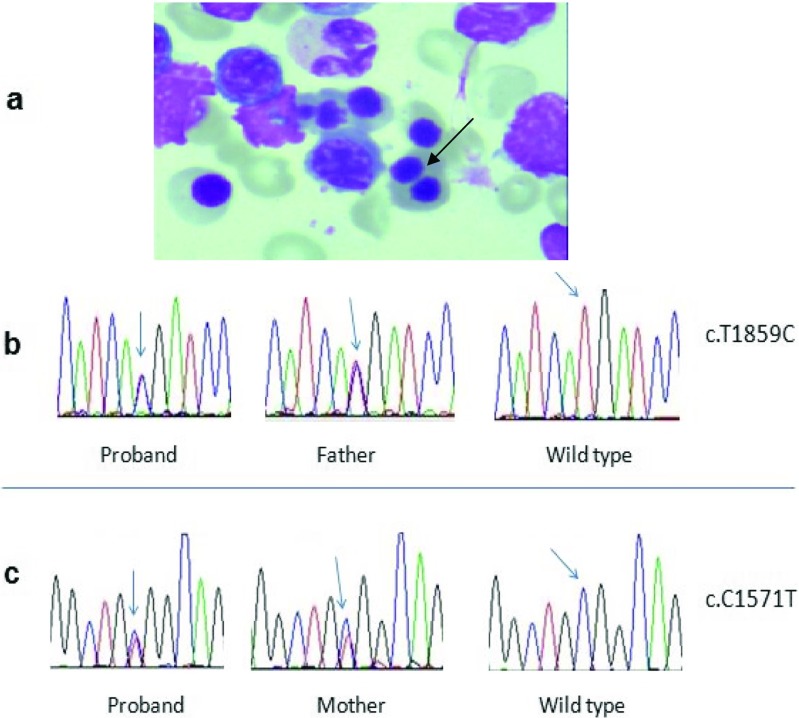
**a** A binucleated late erythroblast on bone marrow smear. **b** Heterozygous mutation (c.T1859C) of the proband and her father. **c** Heterozygous mutation (c. C1571T) of the proband and her mother

Genetic testing of the proband, her little brother, and her parents performed at Shanghai Xin Peijing Medical Laboratory showed two heterozygous changes in the SEC23B gene of chromosome 20, which were heterozygote c.C1571T: p.A524V (on exon 14) and heterozygote c.T1859C: p.M620T (on exon 16). The proband was a compound heterozygote with mutation c.C1571T from her mother and c.T1859C from her father. Her little brother inherited the mutation from their mother (Fig. 1b, c).

In this case, sequencing analysis of CDA-related genes revealed that there were two mutations of SEC23B gene in this family: c. C1571T: p.A524V (on exon 14) and c.T1859C: p.M620T (on exon 16). The proband was a compound heterozygote with mutation c.C1571T from her mother and c.T1859C from her father. Since she was the only patient in this family, the illness of the proband was inferred to have been caused by a compound heterozygous mutation and not by a single mutation. The mutation c. C1571T has been reported [2]. A search of PubMed indicated that the mutation c.T1859C has not yet been reported. Here, we attempted to prove that it was a causative mutation. First, this mutation is located in the gelsolin domain of SEC23B protein, which has an extremely important function. The sequences around this domain are highly conserved among multiple species shown using the Protein BLAST tool. We used the PolyPhen tool to evaluate the possible effects of this mutation. The result was “probably damaging.” Russo [4] reported a CDA II patient whose gene mutation was c.A1858G (on exon 16), adjacent to the mutation site of the proband. Both these mutations resulted in a change in the amino acid at position 620. We believe that mutation c.T1859C is a causative and novel mutation of CDA II.

## Electronic supplementary material

ESM 1 (RAR 2181 kb)
